# Live Tissue Imaging Sheds Light on Cell Level Events During Ectodermal Organ Development

**DOI:** 10.3389/fphys.2020.00818

**Published:** 2020-07-16

**Authors:** Isabel Mogollón, Laura Ahtiainen

**Affiliations:** Cell and Tissue Dynamics Research Program, Institute of Biotechnology/Helsinki Institute of Life Science, University of Helsinki, Helsinki, Finland

**Keywords:** ectodermal, imaging, whole-mount, explant culture, embryonic development, cell cycle, cell migration, cell shape

## Abstract

Embryonic development of ectodermal organs involves a very dynamic range of cellular events and, therefore, requires advanced techniques to visualize them. Ectodermal organogenesis proceeds in well-defined sequential stages mediated by tissue interactions. Different ectodermal organs feature shared morphological characteristics, which are regulated by conserved and reiterative signaling pathways. A wealth of genetic information on the expression patterns and interactions of specific signaling pathways has accumulated over the years. However, the conventional developmental biology methods have mainly relied on two-dimensional tissue histological analyses at fixed time points limiting the possibilities to follow the processes in real time on a single cell resolution. This has complicated the interpretation of cause and effect relationships and mechanisms of the successive events. Whole-mount tissue live imaging approaches are now revealing how reshaping of the epithelial sheet for the initial placodal thickening, budding morphogenesis and beyond, involve coordinated four dimensional changes in cell shapes, well-orchestrated cell movements and specific cell proliferation and apoptosis patterns. It is becoming evident that the interpretation of the reiterative morphogenic signals takes place dynamically at the cellular level. Depending on the context, location, and timing they drive different cell fate choices and cellular interactions regulating a pattern of behaviors that ultimately defines organ shapes and sizes. Here we review how new tissue models, advances in 3D and live tissue imaging techniques have brought new understanding on the cell level behaviors that contribute to the highly dynamic stages of morphogenesis in teeth, hair and related ectodermal organs during development, and in dysplasia contexts.

## Ectodermal Organs, a Versatile Platform for Studying Organogenesis on a Cellular Level

Ectodermal organs arise during embryonic development from the outer layer of the embryo. These organs include teeth, hair follicles, and glands such as mammary, sweat, and salivary glands. This group of organs serve a wide array of different functions from cutting and chewing food, to secretion of saliva or milk, or protection from the elements with hair or feathers. Some of the ectodermal organs have the capacity to constantly grow and renew throughout the lifetime, such as the hairs and the cutting front incisor teeth of mice. Whereas some develop during embryogenesis, but go through extensive re-modification in the adult, such as the mammary gland during lactation. Some ectodermal organs do not maintain a stem cell niche and have lost the renewal capacity. These include the molar teeth of the mouse that develop in the embryonic stages, but then mineralize and erupt after birth and do not grow after that. Unlike humans, that have two sets of teeth the deciduous and permanent teeth, mice only have one. Because of shared developmental features, but also this variety in structure and function, ectodermal organs provide a versatile multifaceted platform to study regulation of embryonic tissue patterning and remodeling in a cell level resolution; the morphogenesis from a flat epithelial sheet to intricate tooth cusp and hair growth patterns and complex ductal branching trees in the glands.

**FIGURE 1:**
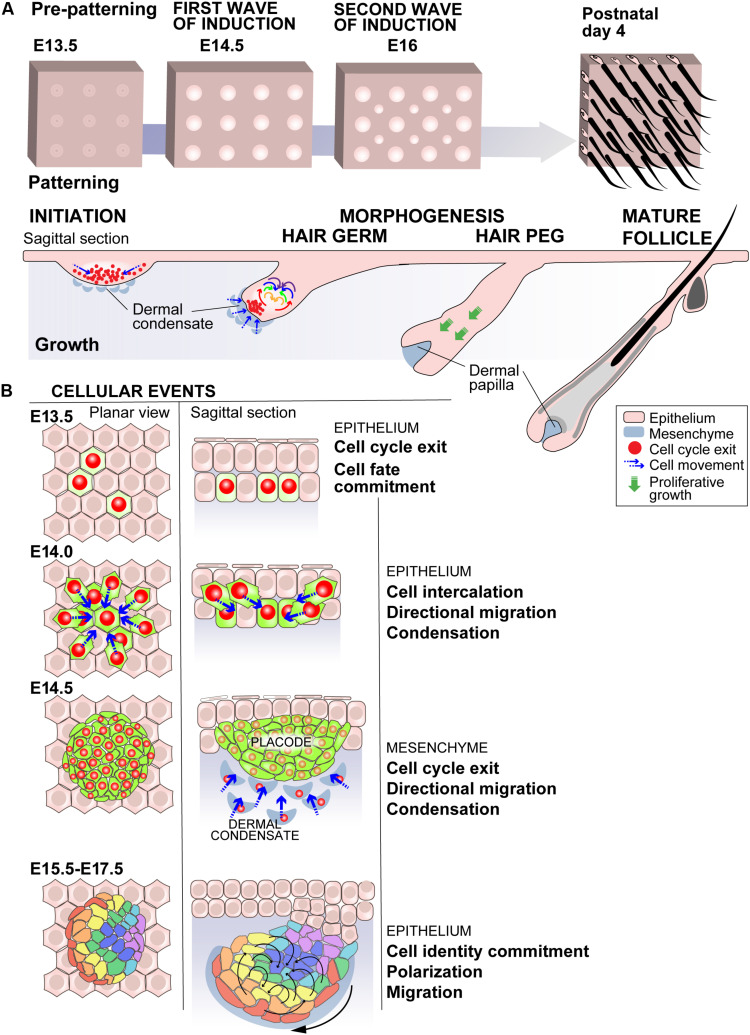
Mammalian early embryonic hair development, a model for cellular mechanisms driving ectodermal organogenesis. **(A)** Mice have four different hair types that arise in three successive waves of hair follicle induction. The first wave of embryonic development of hair follicles in the mouse back skin starts with the establishment of a pre-pattern around embryonic day 13.5 (E13.5) creating the spacing of the hair follicles by a reaction-diffusion type signaling system. At E14.5 the initial thickening of the epidermis, the epithelial placode, is induced by a signal from the mesenchymal compartment the dermis. The epithelium of the hair germ polarizes, and at the same time mesenchymal cells start forming a dermal condensate. The epithelium then buds by cell proliferation invaginating into the dermis to form the hair peg and the condensed mesenchyme engulfed by the epithelium becomes the dermal papilla. This is followed by further differentiation in a mature hair follicle. The second wave of hair morphogenesis takes place around E16–E17 and third at E18- postnatal day 1 (P1) with specific molecular patterning mechanisms for each. The mature hair follicle cycles producing new hairs throughout the adult life. **(B)** The hair has been one of first ectodermal model organs in which live imaging approaches have brought understanding of the cellular dynamics of the morphogenesis and patterning especially in the early stages of morphogenesis: during the first wave of hair follicle induction the cellular events start with a cell fate commitment and cell cycle exit in the prospective placode cells at E13.5. These cells then intercalate and migrate centripetally to condense into a placode thickening at E14.5. The placode signals to the mesenchymal compartment and the dermal cells commit to dermal condensate fate, exit the cell cycle and migrate to form the dermal condensate. The epithelial compartment concomitantly polarizes. The cellular mechanisms of hair follicle polarization have been studied during the second wave of hair follicle induction (E15.5–E17.5). The polarization takes place through planar cell polarity mediated mechanisms and the cells acquire specific fates and collectively actively redistribute into a polarized structure (Duverger and Morasso, 2009; Ahtiainen et al., 2014; Biggs and Mikkola, 2014; Biggs et al., 2018; Cetera et al., 2018).

**FIGURE 2:**
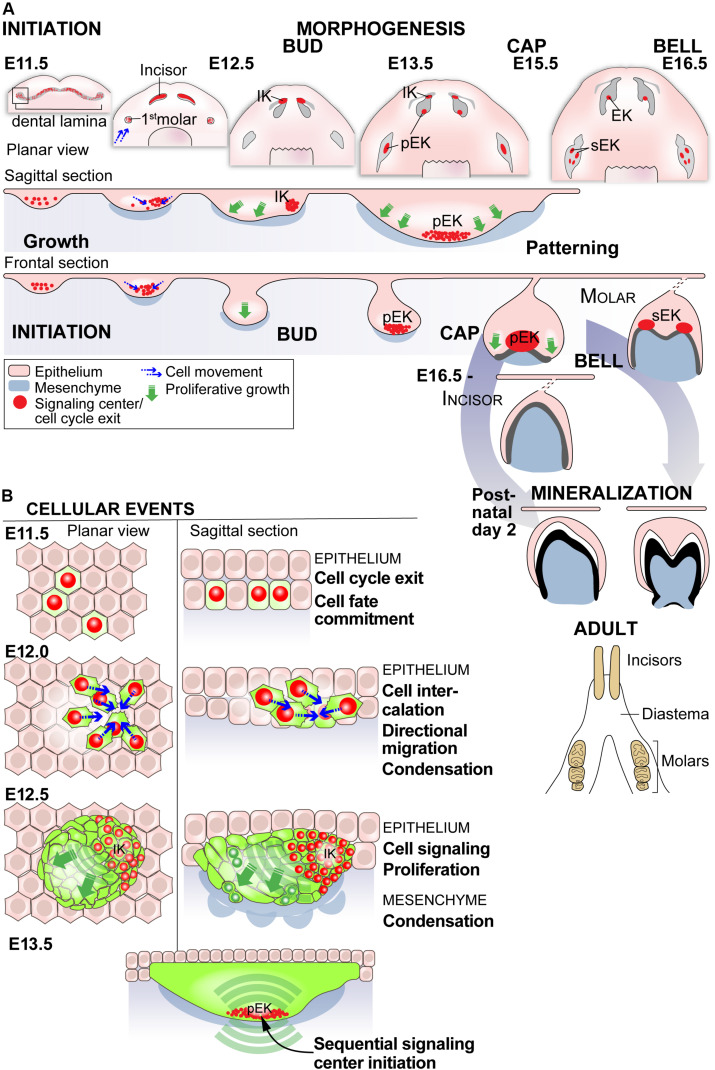
Embryonic development of mouse teeth is governed by specialized signaling centers. **(A)** The embryonic development of mouse teeth in the lower jaw, the mandible, initiates at E11 with a continuous epithelial thickening called the dental lamina. In teeth the initial inductive signal is thought come from the epithelial compartment. The dental lamina resolves into separate placodes for the incisor and molar teeth. A group of cells in the placode acquire a signaling center fate, forming a specialized cluster of cells that signal to the neighboring cells to induce proliferative growth for budding (E12.5). This initiation knot (IK) signaling center is followed by successive signaling centers that use the same signaling pathways reiteratively. The first of these is called the primary enamel knot (pEK) that arises in the bud stage in both incisor and molar teeth. In the following cap stage the epithelium surrounding the signaling center extends deeper into the mesenchyme. Until the stage the development proceeds in a similar way in incisors and molars. The pEK is followed by secondary enamel knots (sEK), but only in the molar tooth, that regulate the epithelial folding into cusp patterns. The first molar is followed by two more molars developing sequentially posteriorly to the first one. In the mouse mandible the incisors and molars have a toothless diastema region in between. The teeth go through differentiation, hard tissue mineralization and erupt postnatally. The incisors continue to grow through the lifetime of the animal, whereas the molars do not grow after complete mineralization. **(B)** The cellular events driving tooth morphogenesis share features with the hair follicle, but there is also differences. The initial epithelial specification into placodes includes cell cycle exit and active cell condensation in both. In the hair follicle the whole placode seems to act as a signaling center, while in teeth a subpopulation of epithelial cells show the hallmarks of the signaling center behavioral signature including specific marker expression, cell cycle exit and active migration for condensation. The epithelial specification is accompanied by mesenchymal condensation also in teeth. The successive epithelial signaling centers, receiving inductive signals from the mesenchyme, shape the developing tooth by controlled ligand distribution driving specific proliferative growth patterns and are ultimately silenced by apoptosis (Jernvall and Thesleff, 2012; Prochazka et al., 2015; Ahtiainen et al., 2016; Morita et al., 2016; Panousopoulou and Green, 2016).

The initial stages of ectodermal organogenesis are very similar in the different organs: They all start out as a simple thickening of the epithelium. Through progressive differentiation and inductive interactions between the epithelium and underlying mesenchyme, they mature into highly differentiated functional organs. The ectodermal organs have for several decades served as model organs of choice to study mechanisms of mammalian embryonic development with respect to tissue interactions and genetic regulation (Jernvall and Thesleff, 2000; Pispa and Thesleff, 2003; Biggs and Mikkola, 2014). The well characterized shared regulatory pathways include Hedgehog, Wnt, Fibroblast growth factor, Ectodysplasin and Bone morphogenetic protein family members (Jernvall and Thesleff, 2000; Biggs and Mikkola, 2014). In recent years, with significant help from emerging new microscopy techniques, the cell level events and interactions within the different tissue compartments are beginning to be understood in detail. These have been challenging to investigate with conventional developmental biology techniques, and also because of the shortage of appropriate reporters to follow the dynamic cellular events in intact tissue. Especially research in the early development of hair follicles (Figure 1) and teeth (Figure 2) using fluorescent transgenic reporter mouse models have paved the way: These studies have led to methodological advances, and identification of hallmark behavioral signatures in specific cell populations and their contribution to morphogenesis. Understanding the delicate balance between proliferative growth, controlled differentiation and cell cycle exit, apoptotic silencing, adhesion remodeling, and cell movement patterns regulated by specific signaling cues in these model organs are shedding light on the fundamental mechanisms of embryonic development.

## Ectodermal Organs Are Emerging as Excellent Models for Live Microscopy to Reveal Cellular Behaviors Shaping the Developing Tissue

Despite shared morphological characteristics and conserved signaling in ectodermal organs (Jernvall and Thesleff, 2000; Biggs and Mikkola, 2014), it is now becoming evident that signaling cues are interpreted into diverse cellular behaviors depending on the context, location, and timing, thereby defining different organ shapes and sizes. Embryonic development proceeds in a dynamic sequence and to properly understand the causalities in the constantly changing system it is necessary to follow the constituents in real time. In recent years, advances in imaging techniques have permitted the field to move from the conventional two dimensional section views to truly three-dimensional (3D) volume and surface data sets and, with live tissue imaging, adding a temporal dimension to the 3D data (4D imaging). These advances together have started to bring new understanding of the cell level behaviors that contribute to the sequential stages of morphogenesis (Sharir and Klein, 2016). Real time visualization of these events is now increasing the understanding of the highly dynamic nature of these processes.

Embryonic ectodermal organs are emerging as models well suited for studies with imaging approaches as well, for several reasons: The development proceeds in morphologically well-defined sequential stages, shared by the different organs, mediated by tissue interactions, and regulated by increasingly well-characterized genetic regulatory pathways (Jernvall and Thesleff, 2000; Biggs and Mikkola, 2014). Transgenic mouse disease models and spontaneous natural mutants, with defects that arrest or alter organogenesis at specific stages, have been widely utilized in ectodermal research. Therefore, the knowledge on specific pathways and their targets and how they can be manipulated genetically is available. Particularly useful is also the pharmacological or mechanical manipulation of cultured explants, together with careful staging of the samples, providing accurate control over the timing of the manipulations (Munne et al., 2009; Närhi and Thesleff, 2010; Ahtiainen et al., 2014; Prochazka et al., 2015; Li J. et al., 2016; Panousopoulou and Green, 2016; Cetera et al., 2018). Finally, the ectodermal organs develop in the surface of the embryo and tissues are highly accessible to imaging with methods that maintain the integrity of the tissue environment.

## Culturing Ectodermal Organs to Maintain the Physiological Environment During Live Imaging

The *in vitro* whole-mount culturing of tissue explants allows the monitoring of growth and morphogenesis throughout specific stages of organ development. Several approaches have been taken to culture embryonic ectodermal tissues for live imaging: the selection of a specific method depends on tissue type, tissue developmental stage, the time span of the process that is being visualized and the available microscopy setups. The advantage of culturing tissues in whole-mount is preservation of an intact environment closely resembling the physiological growth conditions. In the classical Trowell-type organ culture, tissue explants are cultured at the liquid-gas interface on filter membrane supported by a grid (Trowell, 1959). This culture technique supports normal developmental processes in a variety organs in different developmental stages and can be used both for whole-mount tissues or organotypic cultures of dissected tissues and thick sections (Grobstein, 1953; Saxen et al., 1976; Nogawa and Takahashi, 1991; Sahlberg et al., 2002; Cho et al., 2007; Munne et al., 2009, 2010). The classical whole mount tissue Trowell culturing method has been used for live imaging to understand the cellular dynamics of early incisor development in the embryonic mouse mandible and the hair follicle placode and dermal condensate induction in embryonic mouse back skin (Ahtiainen et al., 2014; Ahtiainen et al., 2016; Biggs et al., 2018). Embryonic ectodermal whole mount tissues can also be cultured for live imaging submerged in culture medium mechanically stabilized in the bottom of transparent culture dish. This has been used in the context of early tooth induction where initiation stage (embryonic day E11.5) mandible explants were maintained in a sealed glass bottom dish (Prochazka et al., 2015). For live imaging study of hair follicle development, whole mount back skins (E16.5), during the second wave of hair follicle induction were imaged in an inverted conformation, stabilized between a piece of agarose gel on the dermal side and a transparent gas permeable membrane, forming the bottom of the culture dish, on the epidermal side (Cetera et al., 2018).

The potential challenges with these whole-mount culturing methods include (1) the flattening and spreading of the tissue when removed from the embryo and maintained in culture (2) a slight lag in development caused by culturing (3) increase in apoptotic cells and/or reduction of physiological cell proliferation and increased cell cycle exit especially affecting the superficial layers of the tissue. These issues not only concern the Trowell method, but also the submerged inverted culture as also in this setup tissue needs to be stabilized physically for imaging. It is possible that the artificial distortion caused by the culturing can affect organization of the tissue and possibly also alter cell migration trajectories. All of these effects can be controlled, at least to an extent, by carefully comparing live imaging to the *in vivo* situation with directly fixed tissues of comparable developmental stages.

**FIGURE 3:**
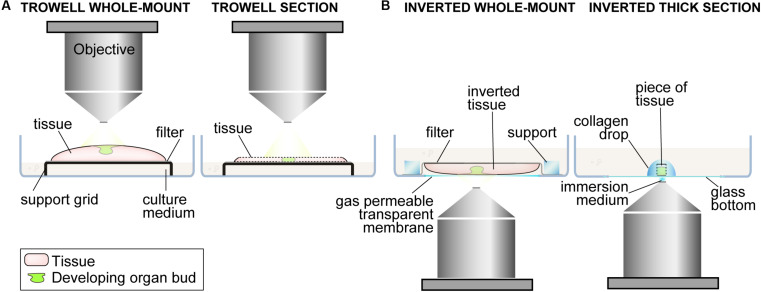
Culturing methods for live tissue whole mount imaging. A schematic representation of culturing setups that have been used for ectodermal live tissue whole mount imaging. **(A)** In the Trowell-type whole-mount setup the tissue, such as the whole embryonic mandible or back skin, is maintained in the interface of air and culturing medium on a filter supported by a grid. The tissue is oriented with the epithelium side upward. This culturing setup allows free gas exchange and maintains the physiologically intact tissue environment. Imaging is done with a upright microscope setup with an air interface objective in an environment controlled chamber. This imaging and culturing setup can also be used with thick tissue sections that are useful especially in later developmental stages when the tissue becomes thicker and/or less transparent and it gives high resolution information in the cross-sectional *z*-axis. **(B)** Tissues can also be cultured for imaging submerged in the culture medium. In this setup the whole-mount tissue is mechanically fixed in place with the epithelium side facing the bottom of the culture dish. The gas exchange and imaging with a inverted microscope setup takes place through a gas permeable optically transparent membrane in the bottom of the dish. The developing organ bud can also be dissected out of the tissue and maintained and imaged similarly.

Especially in later developmental stages, beyond bud stage in the teeth (13.5) and after E16.5 in the back skin, tissue thickness and opaqueness cause limitations to whole-mount imaging. To circumvent this, dissected organs and thick section cultures have been utilized in a variety of setups. In the context of live imaging of molar teeth, from late bud and cap stages (E13.5), Morita et al. (2016) utilized thick frontal slices dissected with needles, containing the central region of the developing tooth and cultured the organ submerged in a drop of collagen. Thick section cultures of molar teeth have been used extensively to study tooth development (Sahlberg et al., 2002; Cho et al., 2007; Munne et al., 2009; Alfaqeeh and Tucker, 2013). In this setup tooth frontal tissue slices (200 μm) are cut with a McIlwain Tissue Chopper (Alfaqeeh and Tucker, 2013). In studies utilizing this method of culturing for live imaging, slices have either been fixed to the bottom of the plate and immobilized using a fragment of coverslip or cultured in a Trowel-type setup (Li J. et al., 2016; Panousopoulou and Green, 2016; Yamada et al., 2019) (Figure 3).

## Microscopy Setups for Embryonic Ectodermal Tissue Imaging

For live imaging, tissue cultures are maintained in a humidified, temperature and pH controlled microscopy chamber that allows non-intrusive confocal fluorescence imaging of the tissue. It is also possible to manipulate specific cellular functions and pathways by adding soluble regulatory molecules or pharmacological inhibitors to the culture medium and follow the effects of cellular behaviors in real time. Live imaging of whole-mount ectodermal organs has been done with an upright microscope setup with an air interface objective or in the inverted conformation with either an air interface objective or water/oil immersion objectives (Ahtiainen et al., 2014, 2016; Biggs et al., 2018; Cetera et al., 2018). The advantages of air interface objectives is the long free working distance allowing accommodation of the tissue and supporting grid/filter or in the inverted setup a gas permeable translucent membrane dish. This type of objective does not suffer from multiple refraction index mismatches, which reduces imaging resolution when imaging live tissues maintained in culture medium with oil/glycerol immersion lenses. However, air interface objectives typically have a low numerical aperture with a restricted light acceptance cone and thus limitations in the light-gathering ability, and suffer from light reflection in the tissue interface and out-of-focus light limiting the achievable resolution (Spencer et al., 2007). Therefore, these imaging setups are best suited for applications where there is high intensity fluorescence signal from the reporters, good signal to background ratio and a need to cover large areas of tissue.

Confocal laser excitation setups that have been used for live imaging of whole mount tissue explant cultures feature either laser scanning or spinning disk confocal microscopes (Ahtiainen et al., 2014, 2016; Prochazka et al., 2015; Morita et al., 2016; Panousopoulou and Green, 2016; Biggs et al., 2018; Cetera et al., 2018). The advantage of a spinning disk confocal is the high acquisition speed. This minimizes the scanning time and with reduced phototoxicity allows acquisition of long time lapses. Laser point scanning excitation on the other hand offers in many cases better depth of imaging, true confocality and is compatible with a weaker fluorescence signal. A disadvantage of spinning disk confocal systems is that the theoretical confocality is somewhat reduced compared to laser scanning systems as some out-of-focus light is transmitted to the camera through closely adjacent pinholes (Conchello and Lichtman, 1994).

Imaging parameters depend on specific sample requirements. These include (1) tissue type in respect to culturing system compatibility, and tissue thickness, tissue opacity and level of inherent autofluorescence, (2) specific fluorescence signal intensity from the fluorescent reporters and signal to noise ratio, (3) length of time lapse, (4) analysis type. In most cases the maximum theoretical resolution has to be compromised with suboptimal sampling in order not to disturb physiological processes and to avoid phototoxicity by extended illumination. Images are acquired as z-stacks, with a sampling frequency in practice roughly a half of the theoretical resolution stated by the Nyquist Sampling Theorem. This translates to 2–5 μm optical sections depending on the analysis target and tissue type (Ahtiainen et al., 2014, 2016; Morita et al., 2016; Biggs et al., 2018; Cetera et al., 2018). Reported stack sampling frequency varies between 5 and 30 min. Optimization depends on sample features. For example detection of multiple fluorophores and/or thick tissues and large areas takes a longer acquisition time decreasing the achievable practical sampling frequency (Pawley, 2006).

## Fluorescent Mouse Reporter Models for Tissue Imaging

Several types of fluorescent reporter models, depending on the application both constitutive and inducible, have been used individually or in combination for live ectodermal tissue imaging (summarized in Table 1). These models can be divided into three, partially overlapping, categories: indicators of cellular behaviors, cell identity/fate, signaling activity. Examples of reporters for cellular behaviors include reporters to visualize cell divisions such as the R26-H2B-EGFP model (Hadjantonakis and Papaioannou, 2004) and the ubiquitinoylation oscillator based fluorescent (Fucci) cell cycle reporters that allow direct real-time follow-up of the progress of the cell cycle in individual cells with a nuclear, dynamically color changing, fluorescent signal (Sakaue-Sawano et al., 2008; Mort et al., 2014). These reporters have also been used to follow cell movements with live imaging. Fluorescent inducible cell membrane bound reporters such as the R26R^mT/mG^ (Muzumdar et al., 2007) and R26R^Confetti^ (Snippert et al., 2010) allow visualization of tissue type, cell shape changes, lineage tracing, and scarce labeling approaches tracking of individual cells. The signaling reporter TCF/Lef:H2B-GFP (Ferrer-Vaquer et al., 2010) is a sensitive fluorescent reporter of canonical Wnt signaling activation and is based on tandem transcription factor binding sites driving expression of the H2B-EGFP fusion protein. Examples of fluorescent reporters for cell identity are the Shh^GFP–Cre^ (Harfe et al., 2004) reporter visualizing signaling center identity, Sox2-GFP for hair follicle dermal condensate cells (Biggs et al., 2018) and keratin 17-GFP to visualize the ectodermal organ epithelium (Bianchi et al., 2005; Ahtiainen et al., 2014).

**TABLE 1:** Fluorescent mouse reporter models for live tissue imaging of ectodermal embryogenesis.

	Mouse model	Characteristics*	Application in ectodermal embryonic research	Live tissue imaging reference
Cell behavior	Fucci (1st generation)	Dynamic cell cycle indicator Ubiquitinylation oscillators emit nuclear red fluorescence in G_1_/G_0_ phase and transition to green fluorescence in S/G_2_/M phases^1^	Cell cycle, migration Hair follicle, incisor and molar teeth	Ahtiainen et al., 2014, 2016; Morita et al., 2016; Biggs et al., 2018
	R26Fucci2aR	Dynamic cell cycle indicator Bicistronic, inducible^2^	Cell cycle, migration Hair follicle	Biggs et al., 2018
	R26-H2B-EGFP	Visualization of mitoses Histone 2B-GFP fusion protein^3^	Cell division Molar tooth	Morita et al., 2016
	R26R^mT/mG^	Constitutive red membrane fluorescence, inducible green membrane fluorescence^4^	Cell shape, movement and lineage tracing Molar tooth, hair follicle	Prochazka et al., 2015; Panousopoulou and Green, 2016; Cetera et al., 2018
	R26R^Confetti^	Inducible random multicolor membrane fluorescence^5^	Lineage tracing Molar tooth	Prochazka et al., 2015
	LifeAct	Fluorescent actin labeling^6^	Cytoskeletal dynamics Molar tooth	Prochazka et al., 2015
	K14EGFP/Actb	Fluorescent actin labeling, under keratin 14 promoter^7^	Cytoskeletal dynamics Molar tooth	Prochazka et al., 2015
Cell signaling	TCF/Lef:H2B-GFP	Active canonical Wnt signaling, nuclear fluorescence^8^	Signaling activity, cell identity, movement Molar and incisor teeth	Ahtiainen et al., 2016
Cell identity	Shh^GFP–Cre^	Cytoplasmic GFP expression consistent with the endogenous *Shh* locus^9^	Hair follicle placode cell subpopulation	Cetera et al., 2018
	Sox2-GFP	Cytoplasmic GFP expression consistent with the endogenous *Sox2* locus^10^	Hair follicle dermal condensate	Biggs et al., 2018
	K17-GFP	Cytoplasmic GFP expression consistent with the endogenous *keratin 17* locus^11^	Ectodermal organ epithelium Hair follicle, incisor teeth	Ahtiainen et al., 2014, 2016
	E-cadherin-mCFP	Cytoplasmic CFP expression consistent with the endogenous *E-cadherin* locus^12^	Ectodermal organ epithelium Molar tooth	Prochazka et al., 2015

*References to mouse models: ^1^(Sakaue-Sawano et al., 2008), ^2^(Mort et al., 2014), ^3^(Hadjantonakis and Papaioannou, 2004)(#006069, Jackson Laboratories), ^4^(Muzumdar et al., 2007) (#007576, Jackson), ^5^(Snippert et al., 2010) (#013731, Jackson), ^6^(Vaezi et al., 2002), ^7^(Riedl et al., 2010), ^8^(Ferrer-Vaquer et al., 2010) (#013752, Jackson), ^9^(Harfe et al., 2004) (#005622, Jackson), ^10^(D’Amour and Gage, 2003) (#017592, Jackson), ^11^(Bianchi et al., 2005) (#023965, Jackson), ^12^(Snippert et al., 2010) (#016933, Jackson).

## Tissue Manipulation in Embryonic Whole Mount Tissue Imaging

### Physical Manipulation

Physical manipulation of a live tissue whole-mount ectodermal organ culture visualized by microscopy, has so far been reported in mouse whole mount back skin cultures with specific laser ablation followed by 24 h culture (Cetera et al., 2018), while Panousopoulou and Green (2016) report the immediate effects of microsurgical manipulation in molar tooth thick section culture.

Cetera et al. (2018) used laser ablation, with a pulsed IR laser, with E15.5 dorsal skin explants expressing ubiquitous membrane-Tomato to test if signals from the mesenchymal dermal condensate are required to maintain cell fate asymmetry in the epithelium in the post-polarization stage hair follicle. Tissues were imaged and cultured in an inverted setup with a 40×/1.0 N.A. water immersion objective. Ablated follicles showed a loss in asymmetry with even expression of cell fate markers, indicating that the maintenance of cell fates requires mesenchymal signals with active repression of stem cell progenitor fate. Tissue welfare and was verified by imaging the tissue immediately after ablation and after culturing.

Panousopoulou and Green (2016) took a different, short term, approach and explored properties of physical tension in the epithelium and basal membrane in slice cultures of placode/early bud stage tooth tissue. They explored mechanisms of epithelial invagination by visualizing immediate effects of microsurgical manipulation. In this experimental setup they made a cut into the epithelium with a needle, directly adjacent to the tooth initiating epithelial thickening. They measured nuclear deformation, and observed tissue shape immediately after the cut, and tissue recoil in the presence or absence of inhibitors of actin dynamics. The frontal slices were maintained immobilized in a bottom of a culture plate and imaging done with a stereoscopic zoom microscope with a real-time viewing (RTV) camera optimized for lowlight time lapse applications. Images were acquired with high frequency every 5sek in a 2 min time window. The condition of the tissue was confirmed form DAPI and fluorescently labeled phalloidin stained tissue after live imaging.

### Modulation of Signaling Pathway Activity

To dissect the molecular mechanisms of ectodermal organogenesis, modulation of signaling pathway activity in the context of live tissue imaging, has been done with administration of pharmacological inhibitors/activators and recombinant proteins, applied either directly into the culture medium or in slowly releasing beads. Prochazka et al. (2015) studied intraepithelial cell migration in the developing mandible. They observed, with whole mount live tissue imaging, a group of fibroblast growth factor 8 (Fgf8)-expressing progenitor cells close to the mandible hinge and followed the actively migrating descendants of these progenitors. The epithelial cell flow was directed anteriorly toward, the initiator of molar tooth development, the Sonic hedgehog (*Shh*) expressing molar placode. To dissect the regulation of this cell movement by different signaling pathways they used pharmacological inhibitors in the culture medium. A wide variety of often redundant Fgf ligands and receptors are expressed during mouse tooth development. Therefore, they used a broad-spectrum inhibitor of FGF signaling the MEK/ERK pathway inhibitor SU5402. Shh signaling was modulated by inhibition with cyclopamine, an inhibitor of smoothened, an ubiquitously expressed transducer of Shh signaling, or by applying a recombinant Shh releasing bead. Live tracking of cell movement showed that inhibition of Fgf signaling impeded cell movement while Shh modulation disrupted movement directionality and cell convergence in this context.

Fgf dependent cellular behaviors have also been studied in hair follicle development. Biggs et al. (2018) studied the cellular mechanisms in the early stages of the mesenchymal dermal papilla formation and the contribution of the dermal condensate specific growth factor Fgf20. They followed normal mesenchymal cell movement patterns during dermal condensate formation with live whole mount imaging of embryonic back skin cultures. They showed that directed migration of fibroblasts drives the condensate formation. This study did not directly address the effects of Fgf modulation on the movement patterns with live imaging. However, they treated explants with both pharmacological inhibitors and recombinant protein bead approaches and observed the effects on cell condensation in fixed tissues. They confirmed the specificity of SU5402 of inhibition: the inhibitor induced a similar stripy hair patterning phenotype to the *Fgf20* mouse mutants (Huh et al., 2013). SU5425 inhibits VEGFR2 and PDGFRB receptors in addition to FGFR1, but blocking these with other specific inhibitors did not block DC formation. FGF20 releasing beads on the other hand, induced DC condensation in explants and cell culture scratch wound and transwell migration assays lead the authors to propose a chemokinetic role for Fgf20 in the DC context.

Ahtiainen et al. (2014) studied the cellular mechanisms in the initial stages of development of the hair follicle epithelial compartment by whole mount live tissue imaging together with the modulation of ectodysplasin/NF-κB and Wnt/β-catenin pathways. They applied pathway activity stimulating recombinant proteins to the culture medium and followed the effect on cell movement and proliferation. The Eda receptor becomes focally upregulated in developing hair placode and offers the responsive specificity to the ligand in this system (Headon and Overbeek, 1999; Laurikkala et al., 2002). Stimulation of the explants with both recombinant Eda and the global canonical Wnt inducer Wnt3a together with a signaling cofactor Rspo2 lead to increased cell cycle exit and increased area of the organ primordium similarly as in the epithelial overexpression mutant of Eda (K14-Eda) (Mustonen et al., 2003). Overactivation of both pathways resulted in increased overall cell motility together with reduced directionality disrupting the centripetal migration for condensation and proper development of the organ.

### Pharmacological Perturbation of Specific Cell Behaviors

Perturbation of specific cell behaviors with carefully timed application of pharmacological inhibitors onto cultured tissues, a method conventionally used in cell biology research in cell culture, can be an precise tool to dissect cell level developmental sequences. Inhibitors applied to cultures affect the whole tissue and do not have the specificity of genetic approaches, where a specific disruption can be targeted to a tissue compartment or group of cells. These limitation in compartment specificity have to be taken into account by employing proper controls and with careful interpretation of the results. Despite this, pharmacological perturbations also have several advantages. Administration into cultures permits accurate control over dosing ranges. Imaging of reporters allows both a very specific developmental stage timing in ectodermal organs for these *ex vivo* treatments and follow-up of both morphogenic and cell level immediate and long term behavioral spatiotemporal effects. Cell motility and shape changes are important cell behaviors in several steps of ectodermal organ morphogenesis. Both depend on cytoskeletal dynamics. The disruption of these has been reported in both development of the hair follicle and teeth with specific small molecule inhibitors, including a myosin II inhibitor blebbistatin, selective Rho kinase inhibitor Y-27632, or toxins such as Latrunculin A (actin polymerization), Jasplakinolide (F-actin capping) (Ahtiainen et al., 2014, 2016; Prochazka et al., 2015; Panousopoulou and Green, 2016; Cetera et al., 2018). Some studies have utilized disruption of cell adhesion in the molar tooth with calcium chelator EGTA (for E-cadherin) or specific inhibitor PF-573228 (for focal adhesion kinase) (Panousopoulou and Green, 2016; Yamada et al., 2019). Also cell proliferation has been targeted: Inhibition of cell proliferation with a DNA polymerase inhibitor aphidicolin in the embryonic back skin did not abrogate hair placode formation or bud-to-cap transition in molar tooth sections, leading to the interpretation that these events do not depend on cell proliferation (Ahtiainen et al., 2014; Yamada et al., 2019).

## Cell Behavior Analyses in Tissue Imaging

Advancements in live tissue imaging techniques are bringing forth a multitude of cell level behaviors and cell population dynamics, within tissue compartments of ectodermal organs. These could not be studied with conventional methods in fixed tissues and 2D sections. Recent research has shown that cell movements are crucial in the morphogenesis and they can only be studied with live imaging. There are different modes of cell movement found in ectodermal organs: these range from long distance large scale rearrangements to local migration within the organ primordia and timescales of days to just minutes. Depending on the specific cell population, movements can vary from directional to random, and homogenous to variable. These patterns depend on the signaling milieu and spatial interactions.

A variety of analyses have been used to dissect the movement patterns in developing ectodermal organs (Table 2). Directionality of cell movement is defined by follow-up of individual cells within the tissue in time and three dimensions and analysis of track orientation (directionality, angle), straightness, length, and persistence. Centripetal cell movement, a specific type of directional movement toward a center point of a circular area, can be identified by measuring escape angles. Cell migration can occur over long distances or in confined areas. An example of a long distance cell flow is the intraepithelial cell migration from a distal progenitor pool to frontal parts of the mandible toward the molar initiating region (Prochazka et al., 2015).

**TABLE 2:** Cell behavior analyses in live tissue imaging of ectodermal embryogenesis.

Cell behavior	Measure	Analysis	References
**Migration**			Ahtiainen et al., 2014, 2016; Prochazka et al., 2015; Morita et al., 2016; Panousopoulou and Green, 2016; Biggs et al., 2018; Cetera et al., 2018
Distance	Track length, net displacement	Movement pattern	
Velocity	Velocity, net velocity	Velocity and variation	
Directionality	Movement angle, track straightness	Orientation, escape angle, directional persistence, variation	
**Rearrangement**			Panousopoulou and Green, 2016; Cetera et al., 2018
Intercalation	Relative position	Cell intercalation	
Convergence		Convergence, collective migration	
**Cell shape**			Ahtiainen et al., 2014, 2016; Prochazka et al., 2015; Panousopoulou and Green, 2016; Biggs et al., 2018
Cell size/shape	Cell size, dimensions	Isotropic/anisotropic change	
Density	Cell density	Cell compaction	
**Cell adhesion**	Junction position/angle	Junction rearrangement	Cetera et al., 2018
**Lineage progression**	Clonality	Lineage tracing	Prochazka et al., 2015
**Cell cycle**			Ahtiainen et al., 2014, 2016; Morita et al., 2016; Biggs et al., 2018
Phase	G_1_/G_0_; S/G_2_/M	Number of cells/ratio/position	
Cell division	Number, orientation	Stratification, growth rate	

Local and directional migration takes place when signaling center cells condense during early tooth development (Ahtiainen et al., 2016). Hair follicle initiating epithelial placode cells and mesenchymal condensate forming cells move centripetally and condense (Ahtiainen et al., 2014; Biggs et al., 2018). Local random cell movements have been described in proliferative tooth bud populations and interplacodal cells in the mouse back skin (Ahtiainen et al., 2014, 2016; Biggs et al., 2018). Local cell rearrangements such as cell convergence, polarization and cell intercalation have been shown in molar teeth and hair follicles in the early stages of organ development (Panousopoulou and Green, 2016; Cetera et al., 2018). Chemokinetic and chemotactic cues direct cell movements and shape changes during epithelial and mesenchymal condensation (Ahtiainen et al., 2014, 2016; Prochazka et al., 2015; Biggs et al., 2018).

Lineage tracing with fluorescent reporters in whole mount tissue culture offers a single cell level resolution, with much higher accuracy than what can be achieved with many conventional methods (Prochazka et al., 2015). Live visualization of cell cycle reporters enables the study of the continuum of relationships among different cell populations in time and space (Table 2).

Large scale analysis of differential proliferation patterns have been done to study dynamic transitions in molar epithelium shape (Morita et al., 2016). Local tracking of individual cells within the organ primordium showed that proliferation is an important mechanism driving tooth budding morphogenesis (Ahtiainen et al., 2014, 2016). In contrast, epithelial signaling center cells in incisor teeth, the hair placode and the mesenchymal dermal condensate cells in the hair exited the cell cycle (Ahtiainen et al., 2016). A summary of analyses of different cell behavior classes with references to ectodermal organ systems is presented in Table 2.

## Imaging Early Hair Follicle Development Reveals Cellular Processes in Early Ectodermal Organogenesis

### Early Hair Development a Success in Ectodermal Live Imaging Analyses

One of the first model organs showing success in imaging cellular dynamics in early embryonic ectodermal organogenesis has been the hair follicle. The development of the hair follicle takes place in defined stages from the initial epidermal placode thickening that signals to induce the condensation of dermal cells. These together form the hair germ that invaginates through epidermal proliferation (Sennett and Rendl, 2012). Embryonic development of the mouse coat hair takes place in three waves, with specific molecular patterning mechanisms for each, giving rise to the different hair types present in mouse (reviewed in Duverger and Morasso, 2009; Biggs and Mikkola, 2014). The spacing of hair follicles is postulated to be regulated by a reaction-diffusion system, while orientation of hair follicles is driven by planar cell polarity (Sick et al., 2006; Wang et al., 2006). Live-imaging studies on whole mount embryonic back skin have revealed the cellular behaviors contributing to initial steps of the first wave of hair placode epithelial morphogenesis, crucial steps in the formation of the dermal condensate in the first wave hair germ (Ahtiainen et al., 2014; Biggs et al., 2018). Live imaging has also been used to study the cellular mechanisms of polarization in the hair follicle during the second wave of hair follicle induction (Cetera et al., 2018).

The cellular mechanisms contributing to the first wave of hair placode induction remained elusive for a long time due to lack of techniques and appropriate reporters to follow the dynamic events on a single cell level in live tissue. The cellular events were first approached, by live imaging in the epithelial context, during the establishment of hair placodes at E13.75. The study (Ahtiainen et al., 2014) utilized embryonic Trowell-type culture of whole-mount back skins and upright laser scanning confocal microscopy of the keratin 14-GFP reporter to visualize the epithelial invagination and quantitative analysis with a fluorescent ubiquitination–based cell cycle indicator (Fucci) mouse to distinguish stages of the cell cycle *in vivo* (Bianchi et al., 2005; Sakaue-Sawano et al., 2008). Prior to this study the interpretation of the cellular mechanisms contributing to hair placode formation, based on expression and histological studies, was very static: basal keratinocytes had been proposed to enlarge and orient vertically to form the thickening (Sengel, 1976) and the contribution of cell proliferation in the early stage prior to downgrowth was unclear. Surprisingly in light of previous hypotheses, live imaging showed that the hair placode is a very dynamic structure featuring cell cycle exit, active cell compaction, and directional centripetal migration. Pharmacological manipulation by inhibition of actin remodeling suppressed placode formation, including specific marker expression and epithelial thickening formation completely. Live imaging together with stimulation of key regulatory pathways, ectodysplasin/NF-κB and Wnt/β-catenin, with application of recombinant proteins to whole mount tissue culture medium showed increased cell motility, suppressed proliferation and induced placodal cell fate. These findings linked cell fate choices and morphogenetic events.

Biggs et al. (2018) later used the same imaging setup to explore the mechanisms of cell condensation in the hair mesenchymal compartment. They studied the dermal condensate, which is the precursor to the dermal papilla, a mesenchymal regulator of hair cycling in the mature organ. A mouse mutant model had shown that the dermal condensate is dependent on Fgf20 as lack of the growth factor resulted in the loss of dermal condensation while the epithelial compartment was able to form placodes (Huh et al., 2013). In the study Biggs et al. (2018) explored how the fibroblasts in the developing skin are regulated by the epithelium derived fibroblast growth factor 20 (Fgf20) from the initial stages of condensation. They combined a transcriptomic approach (RNA sequencing) and live imaging to show that condensation takes place via Fgf20 primed cell cycle exit and cell motility leading to cell aggregation. They imaged the emerging dermal condensate cells from E13.75 for 13 h and used a Sox2-GFP reporter to mark dermal condensate fate and utilized the G_0_/G_1_ cell cycle phase Fucci reporter for cell cycle analysis and cell tracking. They showed with live imaging that the mesenchymal fibroblasts exit the cell cycle and directional centripetal migration of these cells drives the condensate formation.

In the study Cetera et al. (2018) explored mechanisms of epithelial planar cell polarity that define the cell organization and cell fate asymmetry in the invaginating hair bud utilizing whole mount back skin cultures imaged with a spinning disk confocal. The authors used cell tracking with fluorescent reporters, genetic knock out models for *Shh* and planar cell polarity (PCP) components, pharmacological inhibitors and mechanical laser ablation of the dermal condensate. Imaging Shh-Cre driven GFP together with membrane bound fluorescent reporter, at E17.5 during the second wave of hair induction in the back skin, they showed that, again unexpectedly, the polarization of the follicles took place through collective cell movement. The patterns of cell movement were defined by Shh signaling spatially patterning progenitor fates, together with planar cell polarity, and established cell fate asymmetry. Coordinated movement of the epidermal cells induced mesenchymal displacement and the asymmetrical epithelial polarization was further maintained by signaling from these cells. This study showed that spatial cell patterning and polarity within the developing organ can drive collective cell behavior for morphological and cell fate asymmetry.

These studies are prime examples of how ectodermal organ whole-mount tissue culture combined with live imaging analyses of individual cell level behaviors, brings completely new understanding on the mechanisms of embryonic morphogenesis that has not been possible to achieve with conventional developmental biology methods. Imaging approaches have revealed a completely new level of dynamics in the developing skin: The crucial contribution of cell fate changes, coupled to cell behaviors including cell cycle exit and intricately orchestrated cell movement patterns are necessary for the proper progression on morphogenesis and ultimately organogenesis.

## New Understanding of the Cellular Basis of Tooth Development With Live Imaging

### Mouse Teeth as a Model to Study Development With Whole Mount Tissue Imaging

Many of the conventional developmental biology methods that have been used to study the molecular regulation of tooth development rely on static, often 2D, snapshots of the developing tissue. Yet ectodermal organogenesis proceeds in a three-dimensional and dynamic sequence. Following the individual constituents with imaging methods in real time now offers an unprecedented view on the developmental mechanisms and cellular relationships in tooth development. A large part of the understanding of tooth development has come from mouse mutants and studies have mostly focused later stages of development the bud stage and beyond. Whole-mount live imaging approaches are now revealing the cellular mechanisms of initiation and very early tooth organogenesis that have previously remained elusive.

In mice, teeth initiate at embryonic day (E) 11 with a continuous epithelial thickening called the dental lamina. It resolves into separate thickenings, the tooth placodes. At E12.5 the epithelium invaginates forming a bud together with mesenchymal condensation followed by epithelial folding in cap stage (E13.5–E14.5), bell stage and finally hard tissue mineralization (Thesleff, 2016). Mice have two different tooth types: the large ever-growing incisors and multicuspid molars. These are separated by a toothless diastema in between. Tooth morphogenesis is regulated by signaling centers, specialized groups of cells that organize embryonic organogenesis. The best characterized signaling centers in teeth are the enamel knots (EKs). The EKs secrete signals that determine tooth size and shape beyond bud stage. The incisors have only one EK and in molar teeth the EKs arise sequentially. The first, primary EK (pEK), is followed by successive EKs that determine the cusp pattern (Jernvall and Thesleff, 2000).

### Whole Mount Tissue Imaging Reveals an Initiation Knot Signaling Center Regulating Early Incisor Development

Expression of shared molecular markers in tooth placodes and in EKs had suggested the presence of a signaling center regulating tooth development prior to bud stage (Dassule and McMahon, 1998; Jernvall and Thesleff, 2000; Hovorakova et al., 2011). This was first studied with a combination of whole-mount mandible explant cultures and live imaging in the mouse incisor (Ahtiainen et al., 2016) in a similar imaging setup the authors previously utilized in the context of the hair placode (Ahtiainen et al., 2014). This study explored the cellular mechanisms of tooth budding morphogenesis using fluorescent cell identity and cell cycle reporters. The bright and specific fluorescence from the reporters enabled the follow-up of the process for the first time on a cell level resolution in intact tissue. Live imaging, using the Fucci cell cycle reporter, showed that a group of cells within the incisor placode exited the cell cycle. It was specifically these cells that expressed signaling center markers and were identified as a signaling center regulating tooth initiation and therefore called the initiation knot (IK). Whole-mount live tissue microscopy follow-up of the IK cells confirmed that IK cells did not re-enter the cell cycle during budding morphogenesis. Instead, epithelial invagination took place through cell proliferation in the adjacent lingual side cells while the IK was retained close to the epithelial surface. Incisor IK cells exited the cell cycle similarly as in hair placodes, albeit in the hair the whole placode acts as signaling center that is later positioned in the tip of the proliferating invaginating bud (Ahtiainen et al., 2014; Ouspenskaia et al., 2016). Another shared characteristic revealed by tissue imaging approaches was condensation of the signaling cells via active cell migration. The orientation of migration patterns was somewhat different: centripetal and rotational in hair placodes (Ahtiainen et al., 2014; Cetera et al., 2018) versus distal to mesial in the incisor. This suggests either differences in factors and signaling pattern distribution mediating directionality of the movement and/or differences in timing in mesenchymal-epithelial interactions and tissue polarization. Despite these variations cell condensation through active movement is part of the behavioral signature of signaling centers and may be necessary for controlled ligand distribution. Physical condensation has also been shown to cause transcriptional changes for cell differentiation: a study in tooth mesenchyme primary cell cultures showed that physical compression caused transcriptional changes and tooth specific cell differentiation (Mammoto et al., 2011). In another study co-cultures of dental epithelial and mesenchymal cells showed that condensation is necessary for signaling center function (Li C. Y. et al., 2016).

### Whole Mount Tissue Imaging Brings Clarity to the Debate Over Competing Hypothesis Over the Control of Initial Stages of Tooth Development

Previous studies utilizing 3D reconstructions of histological sections had interpreted the signaling center marker pattern, with two temporally distinct expression domains, as evidence of transient rudimentary teeth preceding the actual incisor bud (Prochazka et al., 2010; Hovorakova et al., 2011). Using an epithelium specific fluorescent reporter (keratin 17 GFP) and the Fucci cell cycle reporter (Bianchi et al., 2005; Sakaue-Sawano et al., 2008) to identify signaling center cells, Ahtiainen et al. (2016) showed that both expression domains are part of the same incisor bud. The second important question addressed by this study with live tissue imaging was the relationship of the IK and EK cells. For this purpose, the Fucci reporter was used together with a canonical Wnt signaling reporter [TCF/Lef:H2B-GFP (Ferrer-Vaquer et al., 2010)] to visualize signaling center cells. IK cells were followed until the emergence of the EK revealing that the two signaling centers formed in separate parts of the same bud and were clonally distinct. This was later confirmed by a conventional lineage tracing approach (Du et al., 2017).

The classical view of tooth development, largely based on molecular studies, states that each placode gives rise to a respective functional tooth (Jernvall and Thesleff, 2000). Incisor initiation appeared to follow the classical view in which the placode and successive, clonally distinct, signaling centers regulate morphogenesis driving proliferation for elongation and invagination of the bud (Ahtiainen et al., 2016; Du et al., 2017). The initiation, positioning and invagination of the molar tooth seemed more complicated: Depending on the experimental setup studies lead to seemingly contrasting interpretations about the contributing cellular mechanisms. Histological studies with carefully staged embryos had led to the interpretation that transient epithelial thickenings in the diastema, anterior to the first molars, would be remnants from vestigial rudimentary teeth lost during mouse evolution (Prochazka et al., 2010; Hovorakova et al., 2011, 2013). On the other hand, a study using whole mount mandible cultures and live imaging to explore the mechanisms of the first molar development and positioning (Prochazka et al., 2015), had reported intraepithelial migration along the mesiodistal axis toward the site of molar initiation, the *Shh* expressing placode. Studies in cultured molar sections highlighted epithelial stratification enriched in the placode through vertical cell divisions and short term imaging revealed convergent migration and intercalation of suprabasal cells shaping emerging buds (Li J. et al., 2016; Panousopoulou and Green, 2016). Long term live imaging starting form bud stage up to bell stage cultures of dissected molar teeth (omitting the most mesial and distal parts), and quantification of cell trajectories, divisions and 3D growth landmarks in the epithelium highlighted the importance of differential cell proliferation (Morita et al., 2016). Proliferation in bud to cap transition was similarly highlighted in *in silico* simulations of morphogenesis (Marin-Riera et al., 2018), whereas experimental work on section cultures emphasized cell shape changes over differential proliferation (Yamada et al., 2019).

Especially in the molar, studies with different experimental conditions and approaches seemed to highlight different aspects of the contributing cell behaviors. This is likely reflective of several factors: variable contribution of physical forces including differential adhesion and tissue mechanical properties; the spatial signaling milieu with proper proximo-distal, lingual-labial and vertical (epithelium-to-mesenchyme) signaling gradients; presence/absence of the contributing cell populations.

### Contribution of Cell Proliferation to Tooth Development – A Cell Behavior Sensitive to the Experimental Environment Complicates Interpretations

The exact contribution and timing of cell proliferation to tooth morphogenesis during the initial invagination, and later in bud-to cap transition, has been debated; based on studies in whole mount and section models interpretations have varied. The study by Li J. et al. (2016) used cultured molar frontal sections and pharmacological manipulation of Shh and Fgf signaling. This experimental setup highlighted epithelial stratification in early invagination over cell proliferation. This culturing technique preserves the milieu for epithelial to mesenchymal interactions, but disrupts signaling gradients within the epithelial plane especially affecting planar signaling within the epithelium in the mesiodistal axis. Later whole mount live imaging studies using the Fucci cell cycle reporter showed, that in the incisor, while the placode cells exited the cell cycle and condensed, invagination and bud elongation happened through cell proliferation (Ahtiainen et al., 2016). In the every early developmental stages the signaling, signaling center condensation, and IK driven cell proliferation likely depend largely on planar signaling within the epithelium while signaling depends more on the epithelium-mesenchyme axis at the following developmental stages. Molar frontal slice cultures compromise the mesiodistal axis and omit the mesial IK, likely to affect more morphogenetic events involving these.

In comparison to cultured tissue sections, whole mount tissue culture in many cases better preserves physiological conditions by maintaining tissue interactions, associated signaling, and maintaining physical forces within the tissue. Whole mount tissue imaging studies in later stages of development are, however, limited by tissue size and opacity. The work by Morita et al. (2016) used an intermediate organotypic model of molar tooth development to study the contribution cell proliferation to tissue deformations defining tooth shape in bud-to cap and cap-to-bell stages. In this model the developing tooth was dissected out of the mandible with the very anterior and posterior parts of the tooth germs cut off, but still preserving the middle part of the organ architecture. All cells were tracked with the aid of nuclear GFP-labeled histone 2B (H2B) and the authors quantified cell trajectories, orientations of cell divisions and 3D growth landmarks. With these techniques they showed how spatiotemporal patterns of growth, division, and motility are systematically regulated during epithelial deformation from bud stage up to bell stage. This study highlighted cell proliferation as the main driving force for tissue growth. This idea was supported by a *in silico* modeling study in the same developmental stage (Marin-Riera et al., 2018) and also consistent with findings in the earlier stages of tooth morphogenesis (Ahtiainen et al., 2016). There has also since been a contradicting report in cultured sections (Yamada et al., 2019) suggesting a secondary role for proliferation and emphasizing differential anisometric cell shape changes as the principal mechanism in epithelial deformation. Taken together, some cell behaviors in embryonic ectodermal organogenesis seem to be more sensitive to specific experimental conditions and this should be taken into account when making interpretations.

## Concluding Remarks

The embryonic development and morphogenesis of ectodermal tissues is coordinated by signaling networks that drive intricate programs of interactive cell behaviors within and between tissue types. 4D imaging of the tissue, in physiologically intact configurations for specific processes, is bringing mechanistic understanding on how the molecular networks control cell level actions in the constantly changing dynamic chain of events. Tightly spatiotemporally regulated processes of cell movement, differentiation, proliferation, adhesion, polarization, and apoptotic cell death shape these organs from a simple thickening of the epithelium to highly diverse functional organs.

A lot of the pioneering work in this field especially in the early stages of organ development, that we have reviewed here, has been done in the developing mouse hair follicle and teeth. The mammary gland is also emerging as organ of choice for developmental imaging studies. While emphasis has been more in the branching morphogenesis of this organ, and also another branching ectodermal organ the salivary gland, it will be of interest to see if the early stages of development in these organs follow a similar program of cell behaviors established by live imaging approaches in teeth and hair.

The fundamental understanding of the molecular basis of ectodermal development comes from mouse mutants. It will be of interest to deeper address the cell level impacts in these mutant models with imaging approaches. Especially of interest is the combination of mutant models crossed with fluorescent reporter models and analyzed with new transcriptomic analyses that are applied to whole tissues, such as the spatial genomic analysis method (SGA) described by Lignell and Kerosuo (2019). This will allow correlation of cell behavior and molecular regulation in the developing tissue with high sensitivity even of low expression levels targets. Moreover, the method allows prediction of gene relationships within individual cells. This is promising for bringing detailed understanding of the single cell level regulation of behaviors in different interacting cell populations. Accumulating data from new high throughput transcriptomics studies on different stages of ectodermal organ development will serve as a driving force for new hypotheses on the cell level mechanisms and regulation, that can be further functionally tested with live imaging approaches.

## Author Contributions

IM and LA contributed to conceptualization, writing, review, and editing the text. LA contributed to acquisition and supervision. Both authors contributed to the article and approved the submitted version.

## Conflict of Interest

The authors declare that the research was conducted in the absence of any commercial or financial relationships that could be construed as a potential conflict of interest.
